# Exploring the medical cannabis prescribing behaviours of New Zealand physicians

**DOI:** 10.1111/dar.13476

**Published:** 2022-05-23

**Authors:** Rachel Manoharan, Joya Kemper, Jenny Young

**Affiliations:** ^1^ School of Biological Sciences University of Auckland Auckland New Zealand; ^2^ Department of Management, Marketing and Entrepreneurship University of Canterbury, UC Business School Christchurch New Zealand; ^3^ Plant and Food Research Ltd Auckland New Zealand

**Keywords:** medicinal cannabis, personal construals, prescribing behaviour

## Abstract

**Introduction:**

Many countries are changing their regulations for prescribing medical cannabis. As gatekeepers, physicians significantly impact patient access to cannabis treatments. It is important to explore how physicians view prescribing cannabis in terms of their existing beliefs, knowledge, possible concerns and personal perceptions.

**Methods:**

Individual, semi‐structured telephone interviews were undertaken with 14 New Zealand physicians from various specialties. The interviews were thematically analysed using a phenomenological approach.

**Results:**

The physician–patient relationship was of extreme importance in making prescription decisions, driven largely by trust in the patient. Barriers to prescribing included concern over possible side effects, the quality and standardisation of medication, uncertainty about indications and equity concerns from the high cost for lower socio‐economic patients. Some physicians held concerns over their liability and risks to their reputation if issues arose for patients.

**Discussion and Conclusion:**

The way physicians regard prescribing medical cannabis is based on their personal beliefs and knowledge built up over their medical career. It is important that these are taken into consideration in the design of future guidelines to help alleviate uncertainties and reduce barriers for informed prescribing. While our research and previous research find that physicians generally will follow clinical guidelines based on institutional logics (i.e. the standardised approach to medicine), we find that physicians often allow their personal construals to determine their perceptions and prescribing behaviour to a considerable extent when they practice medicine. Our findings have implications for Continuing Medical Education, marketing and regulation for medical cannabis, especially about the wording of guideline adherence.

## Introduction

The use of medical cannabis has been changing rapidly around the world, with New Zealand one of the latest countries to change its regulations regarding its prescription. Although the general public's attitude and acceptance towards medical cannabis are growing, this is not necessarily reflected within the medical community [1, 2]. As such, the prescription rates for medical cannabis across numerous countries, including the UK, Canada, Australia and New Zealand, remain extremely low as its therapeutic benefits continue to be highly contested [3, 4]. Therefore, it is important to gauge how physicians view prescribing cannabis in terms of their existing beliefs, knowledge, possible concerns and personal perceptions. As gatekeepers to medical cannabis, physicians can significantly impact patient access to cannabis treatments. For example, Oldfield *et al*. [5] found that patients were open to discussing cannabis with health‐care professionals, yet few followed through with the discussions due to concerns around stigma and difficulties in raising the subject.

### Medical cannabis in New Zealand

Research into prescriber perceptions of medical cannabis in New Zealand is particularly important due to recent legislative and administrative changes. New Zealand has one of the highest cannabis use rates in the Western world (15% reported past‐year cannabis use) [6]. Cannabis usage in the New Zealand context is historically nuanced because cannabis is embedded in the “Kiwi” culture, with nearly half of all adults having tried it [7]. Also, the lack of access to medical cannabis puts some patients in the position of seeking illicit cannabis [8]. The *Misuse of Drugs (Medical Cannabis) Amendment Act* was passed on 11 December 2018 (came into effect on 1 April 2020), giving patients in palliative care the right to possess and use medical cannabis [9]. This Medical Cannabis Scheme granted all physicians the authority to prescribe cannabidiol (CBD) and (with ministerial approval) tetrahydrocannabinol (THC)‐containing products. The government also founded a Medical Cannabis Agency to regulate cannabis cultivation, production and prescription [9]. In a recent survey, only half of the previous users of cannabis for medicinal purposes discussed medicinal cannabis use with their medical professional, and a minority requested a prescription [8]. However, 66% of previous users of cannabis for medicinal purposes were intending to transition to the new prescription Medicinal Cannabis Scheme [10]. As these changes are recent, and because physicians are gatekeepers to medical cannabis, it is important to understand what informs their prescription decisions to improve patient access and industry standards moving forward.

Regarding the regulation of medicinal cannabis in New Zealand, prior to April 2020, general practitioners could only prescribe CBD products that contained <2% THC. Only specialist physicians were permitted to prescribe medicinal cannabis products containing >2% THC without ministerial approval [11]. Medicinal cannabis products are comprised primarily by the chemical compounds CBD and THC [12]. While they have the same molecular structure slight differences in the way the atoms are arranged in CBD and THC result in differing effects and interactions with the body's endocannabinoid system (i.e. THC binds to CB1 receptors causing euphoria) [12]. Since 1 April 2020 all physicians may prescribe any medicinal cannabis product that meets the medicinal cannabis minimum quality standards without specialist recommendation or Ministerial approval (refer to ‘Medicinal cannabis products that meet the minimum quality standard under the Misuse of Drugs [Medicinal Cannabis] Regulations 2019’ for products). PHARMAC, the New Zealand funding agency, decides which medicinal cannabis products to subsidise for the community and public hospitals. The Medicinal Cannabis Agency, operating under the Ministry of Health, as well as Medsafe, remain the predominant sources for regulations, prescribing guidelines and information regarding approved medicinal cannabis products [11]. Prior to the foundation of the Medicinal Cannabis Agency in April 2020, prescribing guidelines were virtually non‐existent and left up to individual physician discretion.

### Prescriber perceptions of medical cannabis

Previous research has examined physicians' knowledge, attitudes and prescribing rates for medical cannabis with most taking a quantitative approach. A recent systematic review on medical students and professionals found that nearly 65% believe in the therapeutic utility of cannabis, and just over 40% reported confidence in their knowledge of its health effects [2]. Other research found that perceived knowledge of the pharmacology and pharmodynamics, for example, [13] and awareness about the legality and regulations of medical cannabis is very poor [14]. Studies demonstrate there is a demand for more clinical research to be conducted on medical cannabis [15]. There is also demand for medical education [16], with 86.2% of medical professionals and students wanting more educational material to be available [2].

Research highlights that physicians' concerns around medical cannabis revolve around several issues. Such issues include not meeting the same level of clinical evidence and usual criteria as other prescription medications [17, 18], its cultivation, composition, reproducibility and contamination [19, 20], use with other medicines [21], adverse side‐effects [22], especially surrounding mental health deterioration [23, 24], as well as addiction [25] and potential abuse [26]. Taking a qualitative approach, Zolotov [18] found that physicians in Israel had held divergent, yet intertwined narratives about cannabis. In comparison to conventional medicine and because of its prohibition, cannabis was seen as a non‐medicine, but because of its palliative care aspects it was also perceived as a medicine. This tension highlights the value of qualitative research and importance of further investigation into medical cannabis perceptions and prescribing behaviours. In addition, most studies have focused on North America and Europe and very little research has been carried out in New Zealand.

Recent research in New Zealand both before and after the regulatory change shows some contrasts between general practitioners (GP) and specialists. Prior to the change, 55% of GPs had been asked to prescribe cannabis and over half were aware of pharmaceutical‐grade preparations [3]. In terms of barriers to prescribing, 50% of GPs indicated they did not prescribe due to insufficient evidence before the regulatory change [3] and 62% of oncology physicians said the same after the regulatory change [27]. However, 52% of oncology physicians indicated they would be ‘very likely’ to prescribe a cannabis product if there was proven efficacy and it was funded by PHARMAC [27]. This was lower than the 84% of GPs recorded before the regulatory changes [3]. The cost of medication (vs perceived benefit) was also a significant barrier to prescribing medical cannabis both before and after the regulatory change [3, 27]. Furthermore, over 50% of oncology physicians also indicated they had an insufficient understanding of the prescribing process [27], versus before the regulatory change when only 14.3% of GPs indicated this barrier [3].

As this research is exploratory, and qualitative research is scarce in this area, our analysis is informed by personal construct theory (PCT) [28]. PCT focuses on the processes of individual meaning‐making and proposes that individuals are able to perceive the same experience in different ways according to their unique construals [28]. PCT was chosen for its ability to provide understanding of diverse personal constructs and core values that help individuals make sense of, integrate and interpret phenomena, experiences and events. The impact of personal construals on physicians' behaviours is more likely to occur in contested arenas such as medical cannabis prescription. As Zolotov *et al*. [18] found, experiences are drawn on by practitioners and influence their perceptions of medical cannabis. In addition, O'Rourke *et al*. [29] suggest that social influences and the norms of health‐care professionals need to be considered when examining the determinants of prescribing medical cannabis.

### Research objectives

The research explores the complex physician–patient relationship and focuses on the knowledge, concerns and considerations when considering prescribing medical cannabis. The overall research question is, what informs and shapes physician's prescription decisions regarding medical cannabis? The study builds on previous qualitative research conducted in Israel which showed physicians did not have a consolidated perception about the product [18]. The research provides several recommendations for policy and education, namely, suggestions to address physician concerns about prescribing (i.e. side effects, cost) and the need to foster trust in the physician–patient relationship.

## Method

### Participants and recruitment

Because of the exploratory nature of the research, purposeful recruitment was adopted to strategically recruit participants who could best illuminate the research question [30]. Ethics approval was obtained via the University of Auckland Human Participant Ethics Committee. Fourteen participants were recruited to take part in the interviews. The participants were registered physicians with the Medical Council of New Zealand and resided across New Zealand's North and South Islands. Healthcare in New Zealand is largely provided a government‐funded public health‐care system, as well as a smaller private sector that is funded by insurance companies or directly by patients [31]. Primary healthcare is provided by GPs who are private practitioners. They are not affiliated with the public health‐care system but receive a partial subsidy from the government [31].

A database from a medicinal cannabis company and industry contacts were utilised for the recruitment. This database was compiled by collecting contact information pertaining to physicians from numerous organisations across New Zealand who had attended a range of domestic medical conferences. Both recruitment sources were able to offer a range of participants to include both participants who had prescribed medicinal cannabis and those who had not. Recruitment of physicians who had prescribed medicinal cannabis was deemed difficult because a low‐prescription rate exists in New Zealand, which is why the medical cannabis company database was utilised. Two hundred and nine physicians were contacted by email to participate in this study: 14 accepted the invitation to participate and 195 declined or did not respond to the email. The final sample consisted of five females and nine males from a limited range of specialisations. This convenience sampling method provided enough participants to obtain different perspectives. Most participants were GPs as can be seen in Table S1 (Supporting Information). The anonymity of participants was maintained using pseudonyms and all identifying information was omitted.

### Data collection

The method of telephone interviews was selected because it enabled a conversation to take place in a convenient manner, while recognising the time constraints of the profession. A semi‐structured interview guide included questions about the participant's background (i.e. seniority, medical specialty), history with medical cannabis and open questions about knowledge, attitudes and concerns towards prescribing medical cannabis [Note: The first author (a postgraduate student) designed the interview guide during her internship with a medicinal cannabis company (a requirement of the degree) which was then refined by all the study authors. The medicinal cannabis company did not have any influence over the data analysis or reporting of the findings.]. Interviews lasted for 24 minutes on average and were audio‐recorded and transcribed.

### Data analysis

NVivo Pro qualitative software was utilised to assist with analysis. PCT asserts that an individual's personal experiences, beliefs and understandings, shape their perception of reality, which in turn, can shape their behaviours [28, 32]. Our analysis, in accordance with PCT, was focused on uncovering underlying themes which conveyed aspects of personal meaning making. This is of importance when analysing physician prescribing behaviour because, despite the objective approach of medicine [33], the different experiences of different physicians may significantly impact how they proceed with patient care.

The coding process was an iterative process as described by Braun and Clarke [34]. First, transcriptions were read to familiarise with the data and then initial codes were generated through grouping like ideas related to knowledge, concerns and considerations when considering prescribing medical cannabis. Next, the codes were grouped under several themes. To facilitate trustworthiness in this study, the data is richly described by incorporating direct quotes from the transcripts, which allows for the voice of the source to be heard, as well as providing referential accuracy [35]. The first author conducted the coding and discussed the process and findings with the other two authors. Trustworthiness was further established in this study by seeking feedback on preliminary findings and having interpretations cross‐checked by highly experienced researchers [35].

## Results

Several major and interrelated themes were identified in the analysis. The findings first outline differences in the current awareness/knowledge about medical cannabis, before highlighting the importance of the patient/physician relationship and the role of trust. Area of concerns that may be held are then followed by the inclinations towards prescribing and implications on reputation. Table 1 displays the themes. The detailed findings are illustrated with verbatim examples.

**Table 1:** Themes

1. Current awareness and knowledge about medical cannabis
1.1. Need and willingness for education/training
2. Physician‐patient relationships: The importance of trust
3. Areas of concern towards prescribing cannabis
3.1. Concern over side effects
3.2. Quality and standardisation of products
3.3. Indications
3.4. Equity concerns
4. Prescribing inclinations/usage
5. Implications for physicians on reputation

### Current awareness and knowledge about medical cannabis

A key topic of concern for all physicians was the clinical evidence pertaining to safety and efficacy of medical cannabis. Virtually all physicians commented that they believed the level of clinical evidence for medical cannabis was severely lacking. Awareness of the legal, governmental and clinical caveats pertaining to the prescription of medical cannabis varied greatly amongst the participants. While all participants were cognisant that medical cannabis products are now available for prescription, a few were completely unaware of the Ministry of Health regulations and guidelines for prescribing them. What was common amongst most participants, however, was the sentiment that the Ministry of Health regulations guidelines were unhelpful, and guidelines were confusing and difficult to follow. As Steven (GP, 20 years practicing) stated:

‘*I read it when I was looking at the process for applying for THC/CBD combined products and it was just a bit difficult to follow really*.’

Furthermore, some of the participants also expressed that the regulations and guidelines were too broad and lacked sufficient details for a physician to prescribe medicinal cannabis safely and effectively.

Most of the participants who had prescribed medical cannabis products to their patients relied largely on the manufacturer's guidelines and information on the bottle/packaging. Some participants also found these guidelines unclear and did not trust the information provided by the manufacturing companies. Violet (GP, 50 years practicing) and Melissa (GP, 10 years practicing) expressed the need for proper independent guidelines to protect physicians from liability and to protect patients by ensuring only appropriate individuals are prescribed medical cannabis. As there are no (perceived) clear clinical guidelines for prescribing medicinal cannabis due to insufficient research surrounding its safety and efficacy, it is likely that some physicians may not wish to rely on these less robust strategies of liability avoidance, and instead choose not to prescribe medicinal cannabis altogether.

#### Need and willingness for education/training

Many participants expressed that medical cannabis' novelty may be a barrier to its prescription. They believed it will take some time for physicians to learn more and familiarise themselves with prescribing it. Furthermore, numerous participants conveyed that familiarising oneself with a new therapy can be extremely difficult for physicians, as they are often inundated with work and cannot find time to learn about novel therapies. As Jacob (GP, 35 years practicing) put it:

‘*GPs are overworked and poorly paid for the hours that they put in and expecting them to suddenly understand a new topic and to provide that new service is asking a lot*’.

Conversely, a few participants conveyed that physicians tend to be eager to prescribe new drugs due to the excitement surrounding their novelty. Yet, very few participants reported that they had comprehensively explored the literature, clinical data and prescribing guidelines pertaining to medical cannabis. Several more participants, while demonstrating slightly less knowledge, expressed great enthusiasm in learning more about medical cannabis to improve their medical knowledge, as well as to be in a better position to address this issue should it arise with a patient. As Tom (Psychiatrist, 21 years practicing) stated:

‘*If the data is there, its needs to be broadly disseminated, especially for such a politically divisive substance. Because remember it's not an inert substance, we've had years of indoctrination that cannabis is dangerous, so suddenly if there's suddenly research coming out that it is useful, that needs to be spread through a kind of nation‐wide education programme*’.

A few physicians, on the other hand, appeared rather indifferent about learning more about medical cannabis either due to age (near retirement) or lack of belief in the use of cannabis for medicinal reasons. While all physicians are required to continuously update their medical knowledge by earning a minimum number of Continuing Medical Education (CME) points each year, it is at a physician's discretion as to what subjects they will learn about and the delivery format through which they will learn. It may simply be that some physicians were exposed to the idea of medicinal cannabis earlier on in their career or that alternative medicine was an important part of their culture; therefore, they may be more enthusiastic about educating themselves on medicinal cannabis. Similarly, some participants may have had negative experiences with cannabis or found that other areas of medicine are significantly more important in their practice; therefore, they may be less interested in learning about medicinal cannabis.

### Physician–patient relationships: The importance of trust

While all participants stated that they would need to verify there was an appropriate indication and genuine cause before they prescribed medical cannabis, they expressed varying levels of trust in their patients. As such, a physician's relationship with their patient was of extreme importance. Jacob (GP, 35 years practicing) appeared to be the most trusting of his patients having prescribed medical cannabis products to over 1000 patients and expressing:

‘*I try not to judge the patient. I respect their views and try to understand their reasons*’.

Diana (GP, 12 years practicing), however, appeared far less trusting of her patients and was fastidious about differentiating between genuine patients and those who are drugs seekers. Diana expressed she would have no problem prescribing medical cannabis to patients with chronic pain like fibromyalgia and cancer who had tried standard therapies first. However, she would have to verify their medication history to ensure they were being honest about having tried other standard therapies first; she often views people who request pain medication as being drug seekers:

‘*Patients with cancer or like severe arthritis who can't even get up and walk, they actually try to reduce their intake of pain medication—they don't like taking pain medication. The genuine people, like the cancer patients, they don't really want to be dependent on that and they don't mind having a little bit of pain. But the people who can't bear the pain are the drug seekers*.’ (Diana, GP, 12 years practicing)

Therefore, Jacob (GP, 35 years practicing) and Diana may be seen as the two extreme ends of a trust in patients' spectrum, with the other doctors falling somewhere in between. For example, Angela (Pain specialist, 12 years practicing) and Brian (Rheumatologist, 16 years practicing) are both specialists in pain medicine and appear to trust their patients. Their trusting relationship towards their patients may be influenced by their position as specialists, with their clientele mostly made up of patients who have a confirmed medical condition already verified by a GP.

A number of physicians expressed that they cannot measure the severity of pain in patients with pain‐related conditions and must trust what a patient tells them. However, some physicians, like Angela and Brian, also mentioned that they would employ certain safeguards to ensure that only the appropriate patients would receive medical cannabis treatment. Furthermore, several physicians stated that they would be more comfortable prescribing medical cannabis to their long‐term patients rather than new ones. Most of the physicians also stated that they would not, or would be very reluctant, to prescribe medicinal cannabis to individuals who had a history of drug or alcohol abuse in order to minimise prescription to potential drug seekers. However, some physicians believed that CBD products had a very low potential for abuse and were unconcerned with its possible misuse.

Numerous participants expressed that they were wary about potentially being pressured by patients to prescribe medical cannabis for them, even when it might not be appropriate. As Brian (Rheumatologist,16 years practicing) stated:

‘*There is the potential for doctors to be placed in a fairly uncomfortable position where people demand a prescription for cannabis where it may not be suitable*’.

As illustrated in the quote above, several participants stated that they found it difficult to refuse writing prescriptions for medications that patients might request, due to the patient having paid for the consultation and, therefore, feeling that they must oblige their request.

Each physician will have a unique system of personal construals that they will apply to patient interactions based on their professional medical experience as well as their personal life experiences and beliefs [28]. For example, Jacob favoured a very holistic and personalised medical approach. Luke stated that his medical school training placed a strong emphasis on recognising drug‐seeking individuals. Therefore, it would be unsurprising for most physicians to form construals about drug‐seeking individuals and to exhibit some degree of scepticism or lack of trust in their patients if they had similar training. Yet, Steven's 20 years of experience in general practice differs from Diana and he appears far more trusting of his patients. As such, the physician's past experience seems to have a large impact on perceptions ad prescribing behaviour.

### Areas of concern towards prescribing cannabis

#### Concern over side effects

Concern about side effects was a key barrier to prescribing. One of the most common uncertainties raised by participants was that of the potential adverse effects of medical cannabis and the lack of clinical research into these effects. As Melissa (GP, 10 years practicing) stated:

‘*The harmful effects of medical cannabis also haven't really been studied. Because they haven't been subject to rigorous evaluation, we don't know what those effects might be*’.

Concerns were raised by multiple participants about whether the harmful effects associated with recreational smoked cannabis (e.g. nausea, vomiting, impaired physical and cognitive function) also pertained to medical cannabis, and whether they differed with regards to CBD‐only or THC‐containing products. In Oldfield's *et al*. [27] study on New Zealand oncology physicians, just over a fifth indicated concern about side effects and interestingly no GPs indicated this as a barrier in a 2020 study (prior to regulatory changes) [3].

The potential psychiatric effects of medical cannabis were also raised by most participants, with many reporting that they would not, or would be very reluctant, to prescribe it to patients with mental illnesses, or those on psychiatric medications, as several studies have linked cannabis use and THC to schizophrenia. Multiple participants also expressed concern that medical cannabis may exacerbate anxiety and depressive disorders, which was particularly interesting, as a number of participants also listed anxiety and depression and potential indications for which medical cannabis may be suitable. Adam (Psychiatrist, 12 years practicing) was particularly concerned:

‘*I think in general cannabis causes more harm than good in psychiatric conditions. Even CBD, I've had some patients with bipolar disorder who have used CBD oil and have become manic as well. So I think its best left alone with people who have psychiatric illnesses*’.

Cannabis' potential effect of altering brain structure was brought up by numerous physicians. They expressed concern over what the long‐term effects of medical cannabis might be for children and adolescents whose brains are far more neuro‐plastic and susceptible to change than those of adults. Also raised by almost all participants was the issue of uncertainty surrounding lack of research into the potential drug interactions that medical cannabis may have with other medications, especially psychiatric medications.

#### Quality and standardisation of products

Another concern was the quality and standardisation of the medical cannabis products available in New Zealand. Multiple physicians voiced alarm over the limited range of imported medical cannabis products available for prescription and the lack of information available about their composition and quality. Angela (Pain specialist, 12 years practicing) expressed the need for New Zealand to have chemists to verify the content of imported products to ensure their quality and standardisation:

‘*At the moment we have a product from the brand Tilray shipped in from Australia and I don't know where that's grown, I have no idea how their chemists determine what percentage of CBD is in there, I wouldn't even know if it's a legitimate product! It could be snake oil for all we know!*’

Melissa also expressed frustration with governing medical bodies, such as PHARMAC, not being able to distinguish between the different products that are currently available in New Zealand and not being able to recommend one product over another.

#### Indications

Physicians were also concerned about the indications for medical cannabis. While all participants listed indications that they believed medical cannabis may be suitable for, many of them expressed uncertainty about whether they were in fact appropriate indications for cannabis treatment, and whether CBD‐only or THC‐containing products would be most suitable for prescription.

Much of this uncertainty stemmed from the current state of the clinical research and difficulties that arise when trying compare and replicate studies involving medical cannabis. Melissa (GP, 10 years practicing) expressed concern that different studies look at different indications, use different products and measure different outcomes, making them difficult to compare and discern which products are suitable for which indications:

‘… *studies are not easy to compare as they are looking and different indications, the way they document side‐effects are different, the outcome is different, the delivery of the products are different, and the actual product is different. Some people are looking at whole cannabis extract while other people are looking at THC and some people are looking at CBD*…’.

Several participants reported that they had researched guidelines and recommendations for prescribing medicinal cannabis but found that the information simply did not exist because cannabis has no clear indications.

#### Equity concerns

Last, a key concern was the high cost of medical cannabis. All participants believed the current price of medical cannabis was prohibitively high and a major barrier to their prescription. Angela (Pain specialist, 12 years practicing) illustrated how much medical cannabis typically costs:

‘*For a 25 mL bottle, that's around $200 and it will last you maybe 20 days. Well, actually, if you're doing 1 mL twice a day with a 25 mL bottle, it's not going to last you longer than 12 ½ days! To be honest with you it is fraught*’.

The socioeconomic aspect of patient accessibility to medical cannabis was brought up by multiple participants who expressed dismay at this issue of equity. Numerous physicians stated that they would be amenable to prescribing medical cannabis to their patients, despite the current level of clinical evidence and uncertainties, but that their patients simply cannot afford it. Steven (GP, 20 years practicing) also mentioned that the high price, coupled with the current level of clinical research, made him wary about prescribing it out of fear wasting a substantial amount of a patient's money on a treatment that may not work. Several participants expressed that funding and regulation by PHARMAC was the only means to ensure that patients receive legitimate products, legally, for medical purposes.

### Prescribing inclinations/usage

Despite the near consensus that there is insufficient clinical data pertaining to medical cannabis, five of the participants had still prescribed medical cannabis products to their patients. A sixth participant had prescribed medical cannabis as a script from a previous, trusted doctor. With the exception of Jacob (GP, 35 years practicing), all these physicians had prescribed medical cannabis to patients who did not respond to standard treatments at the patient's request, although they differed somewhat in their decision‐making process. Angela, Brian and Steven appear to have incorporated some elements of holistic, personalised medicine, as described by Mannion and Exworthy [36], into their practice by allowing their patients to have an active role in the formulation of their treatment plans and by demonstrating openness to prescribing medicinal cannabis on a case‐by‐case basis.

The other physicians, who had never prescribed medical cannabis before, expressed that they were still amenable to prescribe it in the future under some conditions. All physicians asserted that they would be more than happy to prescribe CBD‐only products for the treatment of specified illnesses if they were aware of ‘sound’ clinical data and as a ‘last’ resort. This is because they expressed being far more comfortable with CBD's non‐psychotropic nature and believed that it had fewer potential adverse effects compared to highly psychoactive THC. Yet, Luke considered medicinal cannabis as simply a ‘tool’ in the toolbox of therapy modalities, while Violet believed that it might be useful in conjunction with standard early line therapies. Also, Henry's desire to integrate medicinal cannabis as an earlier therapy to mitigate opioid addiction reflects the wider medical community's growing interest in its potential ability to combat the opioid crisis, with several studies having been conducted to explore its feasibility, although with somewhat mixed findings [37, 38]. A few participants expressed that they would be more comfortable with prescribing CBD‐only products, rather than opioids and benzodiazepines, to patients with pain‐related indications, believing that CBD presented fewer health risks and long‐term ramifications than those medicines.

### Implications for physicians on reputation

Physicians prescribing behaviour was not only influenced by their knowledge, trust in the patient and concerns around medical cannabis but also the implications for themselves (i.e. their reputation) of prescribing medical cannabis. Several participants expressed apprehension surrounding the consequences to their reputation that may occur if they were to begin prescribing medical cannabis. The most common apprehension was that should they begin prescribing medical cannabis, it may become widely known in their communities and result in an influx of patients requesting prescriptions for it. As Daniel (GP, 30 years practicing) stated:

‘*Some GPs will say: “Hang on a tick, I'm seriously busy already and if I'm known as somebody who prescribes this then I am going to be inundated with inappropriate requests that are going to be difficult to handle. That's too stressful and I don't want that in my life*”’.

Some participants also mentioned that they were concerned about what being a medical cannabis prescriber might mean for their professional reputation within the medical community. However, there is no research surrounding this area and thus, determining whether or not the wider medical community will in actuality disapprove of physicians who prescribe medicinal cannabis is unknown.

Several participants were highly concerned about the potential negative outcomes that may arise from prescribing medical cannabis, and several participants also raised the issue of how this may affect physicians in terms of liability. As Tom (Psychiatrist, 21 years practicing) stated:

‘*When something is being called a medicine and a doctor is prescribing it, a doctor is then taking responsibility when the patient has an adverse reaction… Where's my responsibility in this? Would I be taken to a medical disciplinary council if something bad happens?*’

## Discussion

This research explored influences on the prescribing behaviour of physicians regarding medical cannabis. The research approach allowed a qualitative and in‐depth ‘lived experience’ of physicians allowing interpretation and understanding of the physicians' perceptions of (non)prescribing of medical cannabis, going beyond statistical and descriptive information found in previous quantitative research. This is especially important in a changing legislative environment as in‐depth interviews allow unexpected findings to emerge [39]. This research finds that knowledge, trust (physician‐patient relationship), concerns and implications for reputation influence prescribing behaviours. We find that physicians develop distinctive, yet unique, personal construal systems which they apply to a wide variety of aspects in their practice of medicine, including their perceptions and prescribing inclinations regarding medical cannabis.

Consistent with the findings of other studies from other countries, physicians described their legislative and procedural knowledge surrounding prescribing medical cannabis and creating dosage regimes to be very poor [e.g. 3, 27]. As such, physicians rely on reinforced internalised guidelines (i.e. when medicinal cannabis is [not] suitable, dosage) which have been informed mostly by tacit sources of knowledge such as their interactions with colleagues, patients, opinion leaders and pharmaceutical representatives, and their early medical training and experience. Previous research demonstrated that over half of New Zealand GPs surveyed were aware of available pharmaceutical‐grade cannabinoid preparations but many are also confused about whether they needed specialist/Ministry of Health approval or PHARMAC funding and there was disagreement about the levels of evidence for the effectiveness of medical cannabis [3]. Indeed, more than half of oncology physicians have reported they have insufficient understanding of the prescribing process [27]. Our research suggests that due to these beliefs, physicians reserve medical cannabis as a last resort for patients who are non‐responsive to standard treatments [e.g. 13, 24]. This was due in part to concerns such as: (i) lack of clinical evidence [e.g. 17, 18]; (ii) adverse side effects [e.g. 22] including interactions with other drugs and impact on brain structure—particularly the highly neuroplastic brains of children and adolescents that are highly susceptible to structural change induced by THC exposure, which can potentially lead to lower cognitive function and poorer outcomes later in life; and (iii) exploitation potential [e.g. 25, 26]; as well as (iv) high cost of medical cannabis—New Zealand GPs find cost one of the largest impediments encountered to prescribing medical cannabis [3, 27].

The research suggests that beyond prescription knowledge, physicians emphasise the physician–patient relationship, highlighting the key role of trust. This is also seen in a shift in viewing patient treatment from what fits the majority (as determined by large clinical studies) to what may benefit each person on a case‐by case basis (i.e. personalised medicine) [40]. This shift is seen and most often achieved by giving patients a more active role in decision‐making processes alongside health‐care professionals, by clarifying acceptable medical options and allowing the patient to choose their preferred course of care/treatment [41]. As such, trust is a big factor in physician–patient relationships [42] and its subsequent impact on medical cannabis prescription. The research suggests that GPs in our study are less trusting of their patients due to usually being the public's first point of medical contact and seeing a wider variety of individuals; while specialists are more trusting of their patients as they see individuals already filtered through GPs. A lack of trust and concern for drug‐seeking individuals may likely be due to their experiences in general practice which may led to inadvertently stereotyping patients. For example, patients directly asking for medical cannabis may be viewed with scepticism (stereotyping), particularly because medical school training has placed a strong emphasis on recognising drug‐seeking individuals [43]. Past research also indicates that a barrier to prescribing medical cannabis is the perceived potential abuse [e.g. 26]. The traditional position of cannabis as an illegal drug in society is interpreted to have a stronger influence on the need for patient trust than for other prescription drugs, due to the perceived stigma that may arise in providing a prescription.

It is suggested that a physician's unique training, professional experiences and personal history mean they develop construals about the areas of medicine they find most interesting, and the way they engage with patients [28]. The impact that patient relationships may have on a physician's prescribing behaviour was apparent in the analysis of personal consturals regarding patient interactions based on their professional medical experience as well as their personal life experiences and beliefs. As an example, Jacob favoured a very holistic and personalised medical approach that respects the values and decisions of the patient. Therefore, his system of personal construals is likely to be strongly informed by his experiences of practicing medicine in this highly holistic and personalised style, thereby making him more likely to trust his patients. This is in sharp contrast to Diana and Luke's relationship with their patients. While scientific or evidence‐based medicine can only recommend treatments that correspond with their proven level efficacy (most often across large patient groups in controlled clinical trials), holistic medicine may recommend treatments that do not have a high level of proven efficacy because they have been reported to work anecdotally on an individual basis [44]. As such, we can see that physicians' personal construal systems are at times shaped, constrained or in conflict with dominant logics that exist within the institution of medicine, particularly, the logic of standardisation [45, 46]. Indeed, the main reason for not prescribing was indicated as insufficient evidence base by 50–75% of respondents [3, 27]. Despite the rigid professional expectation for physicians to adhere to the established institutional logics that shape the medical practice [36] the findings of this study suggest that physicians allow their personal construals to influence their prescribing behaviour more than previous literature suggests.

Our research also indicates that physicians are worried about their reputation if they were to prescribe medical cannabis. Again, this may be interpreted as being due to the unique status of medical cannabis with its association to the illicit drug trade which can lead to a fear of professional judgement if they are seen to be prescribing to patients where other professionals may not see the treatment as warranted. This is similar to patients who report hiding their medical cannabis use to avoid judgement [47, 48]. This worry appears to be linked to the insufficient evidence base for medical cannabis [3]. As there are no perceived clear clinical guidelines for prescribing medical cannabis due to insufficient research surrounding its safety and efficacy, it is perhaps unsurprising that some physicians may instead choose not to prescribe medical cannabis altogether. However, no previous study has identified reputation as a barrier to prescribing medical cannabis. Physicians' personal experiences of over‐work and burnout, or those of their colleagues, may lead them to form construals to determine what their maximum patient capacity and workload is, as well as how to not exceed it. Therefore, physicians may perceive certain actions, like providing new procedures or offering new treatments as high‐risk behaviours that may lead to increased patient numbers, a greater workload, stress and eventually burnout. Although education was seen as a possible way to upskill, the reluctance to self‐educate was strong due to resistance to pre‐conceived notions about medical cannabis. It is recommended that CME on medical cannabis should be well advertised and encouraged in district health boards. It may be that physicians' multiple prescribing behaviours will impact patients who are accessing care and seeking advice (and prescription) about medical cannabis and result in frustration, confusion and illegal possession.

Our research provides several recommendations for policy and education. Given the lack of medical cannabis knowledge, any future guidelines for medical cannabis in New Zealand should be comprehensive yet succinct, be consistent when issued by different medical authorities, have clear dosage or titration regimes and be explicit about any legal intricacies [49, 50]. Previous literature should be consulted to inform future clinical guidelines on the prescription of medicinal cannabis in ways that would make physicians more likely to read and adhere to them. There also needs to be clarity around the prescribing process in the New Zealand context [27]. There were no significant thematic differences between GPs and specialists other than suitable indications for medicinal cannabis, with all psychiatrists conveying that there are no indications (and that there may even be contraindications) for it within their specialisation. This finding suggests that clarity around suitable indications may also be necessary and importantly, as the physicians were unsure about prescription guidelines and dosage (and relying on untrusted manufacturer recommendations), more information must be given about prescription guidelines. Research has shown that the preferred education methods for GPs in New Zealand by a majority is CME sessions, followed by CME online modules and information sheets [3]. Thus, education should preferably be through CME sessions. The Royal New Zealand College of General Practitioners could actively encourage GPs to enrol in courses via their communications platforms to help alleviate unconscious biases and perceived stigma. Lastly, the cost of prescription needs to be clearly communicated (if covered) and PHARMAC must consider the number of medications funded to enable greater access to patients.

The limitations of our study also provide avenues for future research. Our sample is small because of the exploratory and qualitative nature of the study which effects the generalisability of the findings. Future research based on a large‐scale quantitative survey would be of benefit. The generalisability of the findings may also be limited to the sampling method (i.e. organisational database) and future research would benefit from a random sampling approach. Participants were also mostly GPs, with only three specialists being interviewed, meaning the findings will likely not be significant in the context of any specialisations outside general practice.

Future research is needed into medical cannabis to alleviate physician concerns (i.e. side effects, cost) about prescribing and ways of fostering trust in the physician–patient relationship. We recommend that the issue of reputation should be addressed by the cannabis industry to ensure both the social and legal parameters of prescribing medical cannabis are made clear to physicians. In this vein, greater discussion around responsibility should be included in CME. Furthermore, the strategies used to assess drug seeking behaviour should be further investigated in future research. While our research and previous research find that physicians generally will follow clinical guidelines based on institutional logics (i.e. the standardised approach to medicine) [45, 46], we find that physicians often allow their personal construals to determine their perceptions and prescribing behaviour to a considerable extent when they practice medicine. Thus, future research should explore how physicians' multiple prescribing behaviours impact patients who are accessing care (e.g. emotional reductions, accessing the black market).

## Declarations

Human ethics approval was obtained from The University of Auckland.

As the first author was a student completing a degree (Master of Bioscience Enterprise) connection was required with industry. A database from a medicinal cannabis company was utilised for some of the recruitment, but industry contacts were also used. Both recruitment sources were able to offer a range of participants as we wanted to include both participants who had prescribed medicinal cannabis and those who had not. Recruitment of physicians who had prescribed medicinal cannabis was deemed difficult because a low prescription rate exists in New Zealand, which is why the medical cannabis company database was utilised. The student designed the interview guide during her internship which was then refined by all the study authors. The medicinal cannabis company did not have any influence over the data analysis or reporting of the findings; they were merely used as a source of information for data collection.

## Supporting information


**Table S1.** Participant characteristics.
