# Supplementary material for: Exploring the medical cannabis prescribing behaviours of New Zealand physicians
Source: Drug Alcohol Rev. 2022 May 23;41(6):1355–66. doi: 10.1111/dar.13476 (PMC9544511; doi:10.1111/dar.13476)
Supplement: Supplementary file 1 — Table S1. Participant characteristics. [file DAR-41-1355-s001.docx]

| **Name** | **Gender (M/F)** | **Specialisation** | **Years practicing** | **Previously prescribed CBD?**  **(Y/N)** | **Previously prescribed THC?**  **(Y/N)** |
| --- | --- | --- | --- | --- | --- |
| Luke | M | General practitioner | 4 | N | N |
| Melissa | F | General practitioner | 10 | Y | N |
| Daniel | M | General practitioner | 30 | N | N |
| Amy | F | General practitioner | 7 | N | N |
| Brian | M | Rheumatologist | 16 | Y | N |
| Angela | F | Pain specialist | 12 | Y | Y |
| Henry | M | General practitioner | 30 | N | N |
| Steven | M | General practitioner | 20 | Y | N |
| Diana | F | General practitioner | 12 | N | N |
| Tom | M | Psychiatrist | 21 | N | N |
| Jacob | M | General practitioner | 35 | Y | Y |
| Violet | F | General practitioner | 50 | N | N |
| Christopher | M | General practitioner | 8 | Y | N |
| Adam | M | Psychiatrist | 15 | N | N |

Table S1. Participant characteristics
